# Unlocking allelic variation in circadian clock genes to develop environmentally robust and productive crops

**DOI:** 10.1007/s00425-023-04324-8

**Published:** 2024-02-22

**Authors:** Sangam Lal Dwivedi, Luis Felipe Quiroz, Charles Spillane, Rongling Wu, Autar K. Mattoo, Rodomiro Ortiz

**Affiliations:** 1 Hyderabad, 500016 India; 2 Agriculture and Bioeconomy Research Centre, Ryan Institute, University of Galway, University Road, Galway, H91 REW4 Ireland; 3 Beijing Yanqi Lake Institute of Mathematical Sciences and Applications, Beijing, 101408 China; 4 USDA-ARS, Sustainable Agricultural Systems Laboratory, Beltsville, MD 20705-2350 USA; 5 Department of Plant Breeding, Swedish University of Agricultural Sciences, Sundsvagen, 10, Box 190, SE 23422 Lomma, Sweden

**Keywords:** Adaptation, Allelic variation, Biological rhythms, Breeding all season crops, Clock genes and signaling, Heterosis, Non-invasive assays, Stress tolerance

## Abstract

**Main conclusion:**

Molecular mechanisms of biological rhythms provide opportunities to harness functional allelic diversity in core (and trait- or stress-responsive) oscillator networks to develop more climate-resilient and productive germplasm.

**Abstract:**

The circadian clock senses light and temperature in day–night cycles to drive biological rhythms. The clock integrates endogenous signals and exogenous stimuli to coordinate diverse physiological processes. Advances in high-throughput non-invasive assays, use of forward- and inverse-genetic approaches, and powerful algorithms are allowing quantitation of variation and detection of genes associated with circadian dynamics. Circadian rhythms and phytohormone pathways in response to endogenous and exogenous cues have been well documented the model plant *Arabidopsis*. Novel allelic variation associated with circadian rhythms facilitates adaptation and range expansion, and may provide additional opportunity to tailor climate-resilient crops. The circadian phase and period can determine adaptation to environments, while the robustness in the circadian amplitude can enhance resilience to environmental changes. Circadian rhythms in plants are tightly controlled by multiple and interlocked transcriptional–translational feedback loops involving morning (*CCA1*, *LHY*), mid-day (*PRR9*, *PRR7*, *PRR5*), and evening (*TOC1*, *ELF3*, *ELF4*, *LUX*) genes that maintain the plant circadian clock ticking. Significant progress has been made to unravel the functions of circadian rhythms and clock genes that regulate traits, via interaction with phytohormones and trait-responsive genes, in diverse crops. Altered circadian rhythms and clock genes may contribute to hybrid vigor as shown in *Arabidopsis*, maize, and rice. Modifying circadian rhythms via transgenesis or genome-editing may provide additional opportunities to develop crops with better buffering capacity to environmental stresses. Models that involve clock gene‒phytohormone‒trait interactions can provide novel insights to orchestrate circadian rhythms and modulate clock genes to facilitate breeding of all season crops.

## Genes and networks associated with circadian rhythms

**Fig. 1:**
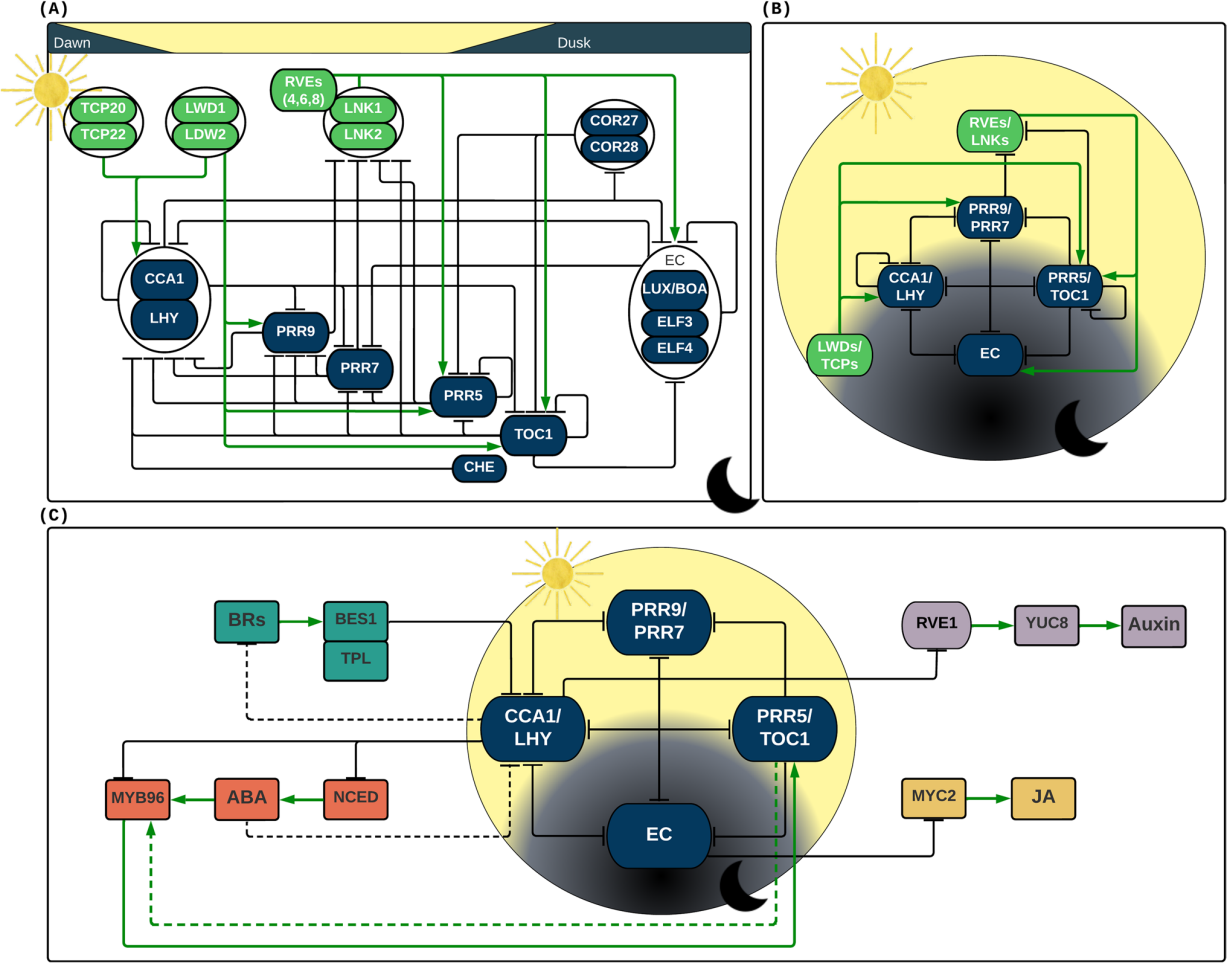
Circadian oscillator components. **A** Extended model representation of the components of the circadian oscillator. **B** Simplified model representation of the components of the circadian oscillator. Solid black lines ending with dashes represent the transcriptional repression. **C** Graphic representation between the hormonal response and the circadian oscillator. The relative timing of action to every component is illustrated in the upper part of the extended model, from left to right during a day–night cycle. Transcriptional repressors are filled in dark blue colour, while transcriptional activators are filled in green colour. Solid lines represent direct regulatory effects while dashed lines represent indirect (or possibly direct but not experimentally determined) effects. Black lines ending with dashes represent the transcriptional repression; green lines ending with arrows represent the transcriptional activation. The circadian repressor components are Circadian Clock Associated 1 (CCA1); Late Elongated Hypocotyl (LHY); Timing of CAB2 Expression 1 (TOC1); Pseudo-Response Regulator 9 (PRR9), PRR7 and PRR5; evening complex (EC) components formed by LUX Arrhythmo (LUX) or Brother of LUX Arrhythmo (BOA), Early Flowering 3 (ELE3), and ELE4; Cold-Regulated 27 (COR27) and COR28; CCA1 Hiking Expedition (CHE). The circadian activation components are Reveille (RVEs); Night Light-Inducible and Clock-Regulated 1 (LNK1), and LNK2; Light-Regulated WD1 (LWD1) and LWD2; Teosinte Branched1-Cycloidea-PCE 20 (TCP20), and TCP22. Proteins involved in brassinosteroids (BRs) circadian regulations are bri1-EMS-Suppressor 1 (BES1) and Topless (TPL). Proteins involved in the Abscisic acid (ABA) circadian regulation are biosynthesis 9-cis-epoxycarotenoid dioxygenase enzymes 3 (NCED3) and MYB96. In the Jasmonic acid (JA) circadian regulation, a basic helix-loop-helix-leucine zipper transcription factor (MYC2) is a key component. Regarding auxin regulation, RVE1 (a member of the RVE transcription factor family but not involved in the core circadian regulation) as well as the auxin biosynthetic gene YUCCA8 (YUC8) are involved

*Arabidopsis thaliana*, a model plant in research, possesses the most extensively characterized circadian network (Fig. 1A and B). Through forward and reverse molecular genetics, it has been elucidated that the circadian rhythm in plants is tightly controlled by multiple interlocked transcriptional–translational feedback loops (TTFL) (Hernando et al. 2017; McClung 2019; Patnaik et al. 2022). A key TTFL is composed of the genes *Circadian Clock Associated 1* (*CCA1*), *Late Elongated Hypocotyl* (*LHY*), and *Timing of CAB2 Expression 1* (*TOC1*). CCA1 and LHY are partially redundant MYB-related Transcription Factors (TFs) that belong to the *Reveille* (*RVE*) gene family, where both are expressed at dawn; i.e. during the onset of the light phase of day–night cycle (Lu et al. 2009; Schaffer et al. 1998; Wang et al. 1997). In contrast, *TOC1* belongs to the pseudo-response regulator (*PRR*) gene family expressed in the evening (Gendron et al. 2012). Both CCA1 and LHY proteins can hetero- and homo-dimerize (Lu et al. 2009; Yakir et al. 2009), and negatively regulate gene expression by interacting with the “Evening Elements” (EE, AAAATATCT) and “CCA1-binding sites” (CBS) on the promoter region of its own genes, of *TOC1* and other circadian clock genes (Carre and Kay 1995; Harmer et al. 2000; Nagel et al. 2015; Wang et al. 1997). TOC1 also acts as a transcription repressor of itself and other circadian regulatory components (Gendron et al. 2012; Huang et al. 2012; Pokhilko et al. 2012). TOC1 represses the expression of CCA1 by interacting with a Teosinte Branched1-Cycloidea-PCE (TCP) protein, the CCA1 Hiking Expedition (CHE) TF (Pruneda-Paz et al. 2009). Thus, CCA1/LHY and TOC1 represent the core negative TTFL in the *A. thaliana* circadian clock, where the morning-expressed CCA1/LHY represses the transcription of the evening-expressed TOC1, while TOC1 inhibits the expression of CCA1/LHY during the evening.

In addition to TOC1, other PRR proteins are involved in circadian rhythm oscillations. PRR9, PRR7, PRR5 and TOC1 are consecutively expressed with moderate differences in their peak expression (Gendron et al. 2012; Liu et al. 2013; Matsushika et al. 2000; Michael et al. 2003; Nakamichi et al. 2010, 2012). PRR9, PRR7 and PRR5 also act as transcription repressors, and together with TOC1, repress the previously expressed PRRs and collectively restrict the transcription of *CCA1* and *LHY* to a period during the dawn (Liu et al. 2013; Nakamichi et al. 2010, 2012). In turn, CCA1/LHY transcriptionally represses *PRR9* and *PRR7* during the dawn (Adams et al. 2015; Fogelmark and Troein 2014; Greenwood et al. 2022; Joanito et al. 2018; Kamioka et al. 2016; Shalit-Kaneh et al. 2018). Another key component of the clock that is transcriptionally repressed by CCA1/LHY and TOC1 is the evening complex (EC) (Gendron et al. 2012; Huang and Nusinow 2016). The EC is composed of the two plant-specific proteins Early Flowering 3 (ELF3) and Early Flowering 4 (ELF4) and the Lux Arrhythmo (LUX), a MYB-like TF that belongs to the GARP protein family or its close homolog Brother of Lux Arrhythmo (BOA) (Dai et al. 2011; Herrero et al. 2012; Nusinow et al. 2011). The EC complex is expressed after dusk and acts as a transcriptional repressor that binds to the promoter regions of *PRR7, PRR9* and *LUX* (Chow et al. 2012; Dixon et al. 2011; Helfer et al. 2011). LUX (or BOA) is the EC component with DNA-binding capability, since ELF3 and ELF4 do not bind to DNA (Huang and Nusinow 2016). Other corepressors involved in the TTFL are COLD-Regulated 27 (COR27) and COR28, which are repressed by CAA1 and repress *PRR5* and *TOC1* (Li et al. 2016; Wang et al. 2017). Nevertheless, COR27 and COR28 cannot bind directly to DNA. Indeed, the TF that interacts with these to repress their molecular target(s) remains unknown (Li et al. 2016).

In contrast to the circadian components described above, the clock also possesses transcriptional activators playing key roles in the TTFL. Unlike CCA1 and LHY, other RVE proteins such as RVE8, RVE4 and RVE6 act as activators of the evening genes by binding to the EE motifs (Farinas and Mas 2011; Hsu et al. 2013; Rawat et al. 2011). These RVEs interact with the coactivators Night Light-Inducible and Clock-Regulated 1 (LNK1) and LNK2 to form a complex and activate the transcription of *TOC1*, *PRR5* and *ELF4* (Rugnone et al. 2013; Xie et al. 2014). Conversely, PRR9, PRR7, PRR5, and TOC1 repress the transcription of *LNK1* and LNK2 from noon to early evening (Rugnone et al. 2013). Other transcription co-activators that play an early role in the circadian oscillations are the Light-Regulated WD1 (LWD1) and LWD2 (Wu et al. 2008). Dawn transcriptional activation of CCA1 requires the recruitment of LWD1 and LDW2 in conjunction with TCP20 and TCP22, two CHE-related transcriptional regulators that belong to the TCP family are able to bind to the TCP binding sites (TBS) in the promoter regions (Mockler et al. 2007; Wu et al. 2016). Several other clock genes have been proposed to recruit LWD1 and LWD2 to their promoter regions to be activated, including *PRR9*, *PRR5*, and *TOC1* (Wu et al. 2008). Despite what has been elucidated to date, the TF(s) involved in recruitment of LWDs to the genes expressed at dusk remains to be determined.

## Circadian clock, the central regulator of plant adaptability

Circadian clocks are drivers of biological rhythms that anticipate the light, dark, and temperature cycles of the rotating Earth. Thus, the circadian clock affects the timing and order of different biological processes and modulates signaling pathways in response to stimuli, which become dependent on the time of day they occur. Artificial selection has led to large changes in the circadian clock of plants through domestication (Siqueira et al. 2023). Indeed, domestication has induced variation in the peaks determining photoperiod perception, whereas changes in the time between peaks has smaller effects, as noted in tomato (Xiang et al. 2022).

Measuring circadian rhythms is a significant challenge in quantifying variation associated with circadian dynamics. The circadian rhythm (i.e., behavioral changes that follow a 24-h cycle) consists of a period (i.e., the time taken to complete one cycle, phase (i.e., the time of day when this peaks), and amplitude (i.e., the distance between the peak and the base line of oscillation). The circadian oscillator refers to networks of biochemical feedback loops that generate 24-h rhythms in living organisms (Saini et al. 2019). Equally important is the rhythm robustness (including resilience after disturbances); i.e., parameter (phase shifts) changes over time.

Circadian clocks help plants to buffer environmental changes through adjustments in their development and physiological characteristics (Sánchez and Kay 2016). *Arabidopsis thaliana* has become the plant model system for extensive investigation to understand how plants continuously adjust their circadian oscillator. The circadian oscillator has a dynamic plasticity (i.e., trait changes expressed by a genotype across different environments (Webb et al. 2019). Circadian clocks in plants are responsive to environmental signals via changes in period and phase. The genes regulating the circadian clock—e.g. transcriptional regulators acting as repressor and activators—control various processes during their development, particularly those related to primary metabolites in the life cycle (Kim et al. 2017). The clock transcriptional network of *Arabidopsis* is partly present in other angiosperms (Nakamichi 2020), which poses challenges to translate results from this model plant to crops.

Selection for early flowering during both domestication led to crop expansion related to photoperiodic adaptation (Lin et al. 2021). Selection of late flowering mutants allows longer growth periods to maximize yield in low latitudes. Short day plants, however, flower later when expanding to high latitudes due to their photosensitivity (Greenham et al. 2017). Various genes [phytochrome red light signaling; circadian clock *ELF3*, *ELF4*, *LUX*, and *Pseudo-Response Regulator37* (*PRR37*), a legume-specific maturity (*E1*), and a short-day monocot-specific *heading date* (*Ghd7*)] contribute to adaptability of grain crops such as barley (*Hordeum vulgare*), maize (*Zea mays*), rice (*Oryza sativa*), pea (*Pisum sativum*), sorghum (*Sorghum bicolor*), soybean (*Glycine max*), wheat (*Triticum aestivum*) (McClung 2021 and references therein) and vegetables, e.g. tomato (*Solanum lycopersicum*) (Xiang et al. 2022). These are putative orthologs of genes found in *Arabidopsis*, which facilitated adaptation of these crops to new latitudes beyond their centers of origin. Osmotic stress, high temperature, light intensity, or total period length, amongst others, can change plant circadian oscillators.

During latitudinal migration, genes related to flowering time were selected in tomato (Blanca et al. 2022). Mutations in two genes (including phytochrome A-specific light signaling gene, *EID1*) were involved in this process. Moreover, deletion of clock gene(s) enhanced crop adaptation in tomato (Müller et al. 2016). Signatures of a selective sweep were detected in the genomic region harbouring *EID1*, which enhances performance under long day photoperiod in tomato. A partial deletion in the clock genes *LNK2* is associated with increasing the photoperiod lengthening in this neutral-day crop (Müller et al. 2018). This example provides information about how humans selected for modulation of circadian rhythms when moving tomato cultivation away from the equator. It appears that mutant alleles in both *EID1* and *LNK2*, which arose in early cultivated tomato and were further selected during its domestication, affected the light input to the circadian oscillator and led to the observed light-conditional deceleration when changing the day–night cycles in the high latitudes. Transcription profiling of near isogenic lines (NILs), homogeneous genetic stocks generated by repeated backcrossing with selection for the desired character at each round of crossing, combining wild alleles of *EID1* and *LNK2* grown under two different photoperiods further revealed that the former had a larger effect on tomato response to daylength (Xiang et al. 2022). This may be attributed to the shift elicited by *EID1* in the circadian rhythm of this vegetable crop. Other clock-associated variants that facilitate crop latitudinal adaptation are related to the regulation of seasonal rhythms during developmental transitions, e.g. flowering and tuberization (Nakamichi 2015). These research findings are useful for breeding to increase crop productivity at high latitudes, particularly in crops with detrimental circadian asynchrony.

## Detecting circadian clock genes

As the basis of an intracellular timekeeping system, circadian clock genes generate approximately 24-h rhythms in physiology and behavior. The detection of clock genes can be based on two complementary approaches. The forward-genetic approach is based on arhythmic mutants that produce rhythms deviating from 24-h cycles. Although this approach has been instrumental for revealing the detailed molecular mechanisms of circadian rhythms in model systems and various outcrossing species (King and Takahashi 1996; Miyoshi et al. 2019), it has some significant drawbacks. First, its translational relevance for a broad spectrum of species and biological processes is challenging since only a limited number of mutations can be identified. Also, many clock genes are not evolutionarily conserved; e.g., some clock genes are species-specific (Calixto et al. 2016). This suggests that ideally, detection of clock genes should be based on individual species. To overcome these drawbacks, a forward-genetic approach has been developed which takes advantage of genome-wide mapping and association genetics available for a range of species (Jones et al. 2019). Such an approach could identify a more complete set of clock genes. For example, using a mapping population, a number of QTL for circadian clock parameters, such as period, phase, and amplitude, were identified in *Arabidopsis thaliana* (Rubin et al. 2019). A review of progress in clock QTL mapping and its application to understanding the genetic architecture of plant adaptation is available (Anwer and Quint 2022).

Most clock mapping attempts have been based on genotype–phenotype association analysis using markers. However, the results from these analyses remain an approximation of reality as the circadian rhythm is complex, involving interactions of many genes with environment. A computational algorithm to map a complete set of clock genes using association genetics has been developed (Sun et al. 2021). This algorithm is powerful in terms of its capacity to reconstruct omnigenic interactome networks for rhythmic traits (Sun et al. 2021). Omnigenic theory predicts that all genes that an organism may carry can be involved in controlling a complex trait developed through gene–gene interaction networks (Boyle et al. 2017). Factoring epistasis interactions (i.e., epiGWAS) could resolve some of the missing heritability for complex traits often found in GWAS or biparental populations (Slim et al. 2020). The algorithm can coalesce all genes into bidirectional, signed, and weighted networks, allowing for the characterization of a roadmap of how each and every gene affects circadian rhythms through direct and indirect pathways. It can also discern how each gene functions. First, some clock genes are significant due to their own strong intrinsic capacity, thus implying that these genes govern circadian rhythms without relying on the promotion of other regulators. Second, the significance of some genes is due to favorable regulation involving other genes. The practical use of these two gene types to improve rhythmic traits should be based on different strategies. In addition, some genes are detected to be insignificant not because their effects are inherently small but because they are inhibited by negative regulators. Thus, the effects of these insignificant genes can be released by knocking down the negative regulators. In theory, this is also an approach for retrieving missing heritability, a common phenomenon detected in current genome-wide association studies. The statistical robustness of this approach has been confirmed (Sun et al. 2021) and also its biological value has been validated by analyzing periodic gene expression data of the malaria parasite *Plasmodium falciparum* (Smith et al. 2020) and growth trajectory data under contrast environmental conditions in a woody plant, *Populus euphrates* (Wang et al. 2021).

## Non-invasive, high throughput assays to measure circadian rhythms

Lack of high throughput protocols has hindered progress in investigating circadian dynamics where such assays were either manually intensive, or of low throughput, technologically expensive, or required genetic modification (Ford et al. 2016; Nagano et al. 2012; Sugiyama et al. 2001; Xu et al. 2010). A non-invasive high-throughput assay based on chlorophyll fluorescence was deployed to measure variation in circadian rhythms among wild barley (*Hordeum vulgare* ssp. *spontaneum*) from diverse ecogeographical environments. Wild barley accessions showed variability for circadian traits. Circadian period lengths were correlated with temperature and site of origin of the plants, while the amplitudes of the rhythms were correlated with soil composition (Dakhiya et al. 2017). Delayed fluorescence (DF) image-based technology is a universal tool to measure circadian rhythms in higher plants (Gould et al. 2009). A further optimization of DF protocol (Rees et al. 2019) led to discovering significant differences in circadian periods between widely grown rapeseed (*Brassica napus*) and wheat cultivars. The latter displayed rhythm robustness under constant light vs constant darkness, thereby suggesting divergent networks underly circadian control in these species.

Plant growth rhythm in structural traits measured by terrestrial laser scanning (TLS) is crucial for better understanding of plant response to the ever-changing environment. TLS has been utilized for studying diurnal rhythms in maize structural traits at both the plant and the leaf levels from time-series data at four key growth periods (jointing, bell-mouthed, heading, maturity) under optimal and cold stressed field environments (Jin et al. 2021). Leaf inclination angle between the jointing stage and well-mouthed stage significantly decreased, with leaf azimuth remaining stable after the jointing stage. A few individual-level structural rhythms were consistent with leaf-level structural rhythms. The circadian rhythms of some traits were diurnal. Environmental factors (particularly temperature) led to better correlations with leaf traits under cold stress than under optimal conditions. This research highlighted the potential of time-series TLS in studying outdoor agricultural chronobiology (Hotta 2021; Steed et al. 2021).

Daily rhythmic movement of leaves has been extensively used to estimate circadian period in *Arabidopsis*. Manual estimates of circadian period by leaf movement is limited to analyzing one leaf or a cotyledon at a time. TRiP (Tracking Rhythms in Plants) estimates rhythm period using a motion estimation algorithm to measure whole plant images. It successfully tracked the movement of cotyledons and leaves of *Arabidopsis* RILs without the need to select individual leaves and could be applied to plant species with diverse leaf morphology (Greenham et al. 2015). A lightweight digital inertial measurement unit (IMU) sensor measures leaf movements and is easily attached to a leaf or plant organ to record angular traits in real time, both simple movement as well as complex lamina motions, for two dimensions with high resolution and detects small changes of mutations in the leaves of varying ages in diverse plant species (Geldhof et al. 2021).

Clearly, significant progress has been made toward dissecting the components of circadian rhythms, making it possible to study rhythmicity and apply this knowledge in plant breeding programs. Further advances are needed to develop protocols adapted to handling early generation breeding populations for selection of segregants with desired rhythms and expressing clock genes.

## Circadian control of phytohormone biosynthesis and signaling shaping plants to external environments

Phytohormones are signaling molecules present in plants that occur in extremely low concentrations and play a major role in plant growth, development, reproduction, and fitness. Abscisic acid (ABA), ethylene (ET), jasmonates (JA), and salicylic acid (SA) are some of the stress-responsive phytohormones, while auxin (AUX), brassinosteroids (BRs), cytokinins (CK), and gibberellins (GA) are growth promoting phytohormones (Gray 2004; Verma et al. 2016). Phytohormones control specific features of the plant–circadian system, though in distinct ways. CK delays circadian phase, AUX regulates circadian amplitude and clock precision, and BR and ABA modulate circadian periodicity. This indicates that plants have multiple input/output feedbacks to adjust the clock to external signals and accurately maintain the clock system (Hanano et al. 2006). The circadian clock interconnects with an intricate network of different pathways, including phytohormones (Singh and Mas 2018).

*Arabidopsis* has been extensively investigated to unlock the molecular basis of connecting the circadian rhythms and phytohormone pathways in response to endogenous and exogenous cues (Fig. 1C). A complex interaction between the circadian clock and ABA pathways in *Arabidopsis* regulates plant responses to abiotic stress tolerance. Late Elongated Hypocotyl (LHY) transcription factor (TF) modulates ABA biosynthesis and ABA responses, which drives the rhythmic accumulation of ABA to ensure peak accumulation of phytohormones when water deficit is most severe. *LHY* gene represses 9-cis-epoxycarotenoid dioxygenase enzymes, a rate-limiting step of ABA biosynthesis. Plants overexpressing *LHY* show reduced levels of ABA under drought, whereas loss-of-function mutants exhibited altered rhythm of ABA accumulation. *LHY* also binds the promoter of multiple components of ABA signaling pathways and regulates responses downstream of the phytohormone. *LHY* promotes the expression of ABA-responsive genes involved in stress tolerance while inhibiting the negative effect of ABA on seed germination and plant growth (Adams et al. 2018).

Circadian clock gates respond to environmental signals to maximize plant fitness. Clock gating is the clock’s regulation of signaling pathways in a manner that the same stimuli given at different time points result in different responses, usually different intensities, which help plants to reduce stress responses at certain times of the day to ensure optimized growth. The MYB96 TF connects with the clock oscillator to shape the circadian gating of ABA responses by directly binding to the *TOC1* promoter to regulate its expression as evidenced in *Arabidopsis myb96* and *toc1-3* mutants. *CCA1* may also regulate *MYB96-TOC1* function to alter its circadian expression. Thus, a complex circuitry of *CCA1-MYB96-TOC1* regulatory interactions provides the mechanistic basis to connect circadian clock and stress signaling to optimize plant defense and fitness (Lee et al. 2016). BRs control many physiological processes via extensive cross-talks with diverse signaling networks. The BR-activated TF *bri1*-EMS-Suppressor 1 (BES1) together with *TPL* represses *CCA1* and *LHY* expression at night by binding to their promoters. This repression by BR treatment is compromised in *bes1-ko* and *tpl8* mutants. Consistent BRs treatment for an extended periods shorten the circadian period, where BR-induced rhythmic shortening was impaired in *bes1-ko* and *tpl8* single mutants as well as in the double mutant, *cca1-1lhy-21*, providing evidence that *BES1/TPL-CCA1/LHY* module shapes circadian gating of BR signaling in plants (Lee et al. 2020).

Leaf senescence is associated with plant ageing or induced by external stresses to relocate nutrients from aging tissues to juvenile and reproductive organs. JA, an endogenous signal, induces leaf senescence (Sobieszczuk-Nowicka et al. 2018). The evening complex (EC), a core component of the circadian oscillator, negatively regulates leaf senescence. EC is involved in JA signaling and response as evidenced by accelerated leaf senescence in EC mutants upon JA induction. EC binds *MYC2* promoter to repress its expression. *MYC2* is the key activator of JA-induced leaf senescence as in EC triple (*myc2 myc3 myc4*) mutants. The accelerated JA-induced leaf senescence in EC mutant is abrogated by *myc2 myc3 myc4* triple mutation, providing direct evidence that a core component of the circadian clock gates JA signaling to regulate leaf senescence (Zhang et al. 2018b). JA signaling regulates multiple outputs of plant defense and growth to minimize adverse effects of growth-defense trade-offs in complex environments (Li et al. 2022).

Auxin plays a central role in plant growth and development in response to environmental cues. *RVE1* is a Myb-like clock regulated TF homologous to *CCA1* and *LHY*. Its inactivation has no effect on circadian rhythmicity but instead causes a growth phenotype, indicating it as a ‘clock output’ that affects plant development. *CCA1* regulates growth via bHLH TFs *PIF4* and *PIF5*. However, *RVE1* acts independent of *PIF4* and *PIF5*. *RVE1* regulates auxin levels by promoting its production during the day but not at night. It positively regulates the expression of auxin biosynthesis gene *YUC8* to promote growth. Thus, *YUC8* connects two signaling networks to coordinate plant growth with rhythmic changes in the environment (Rawat et al. 2009).

*OsCCA1*, a key clock component in rice, confers tolerance to drought, osmotic, and salinity stresses. There are 692 direct transcriptional target genes of *OsCCA1* where, genes involved in ABA signaling are substantially enriched. *OsCCA1* binds the promoters of *OsPP108* and *OsbZIP46* to activate their expression. *Oscca1* null mutants display enhanced sensitivity to ABA signaling. Thus, abiotic stress adaptation by *OsCCA1* is orchestrated by ABA signaling, linking the circadian clock with ABA signaling in rice (Wei et al. 2022). The OsRCAR10-OsABI5-OsPRR95 feedback loop regulates ABA signaling to fine-tune seed germination and seedling growth, indicating molecular link between ABA signaling and circadian clock in rice (Wang et al. 2022a).

## Modifying circadian rhythms to develop climate-resilient productive crop cultivars

### Novel variation in circadian clock systems

The presence of natural variation for circadian rhythms enables plant species to adapt to a wide range of latitudes with variable climates. The examples include variation in circadian period along a latitudinal gradient in annual population of common yellow monkeyflower (*Mimulus guttatus*) and domesticated soybean crop (Greenham et al. 2017); period length covaries with both geography and population substructure among Swedish *Arabidopsis* accessions (Rees et al. 2021); free running period (FRP)-dependent phase phenotype underlies photoperiod (short day) adaptation of Japanese duck weed (*Lemna aequinoctialis*) (Muranaka et al. 2022); and deceleration in circadian clock due to phase delay caused by allelic variation of the tomato homolog of the Arabidopsis *EID1* expanded adaptation of domesticated tomatoes in farthest environment in Europe (long day photoperiod) far away from the center of origin (Müller et al. 2016).

Many crop wild relatives harbour genetic variation which enables them to flourish in harsh environments. Circadian rhythms in wild barley populations from ecogeographical locations of Southern Fertile Crescent in the Middle East, for example, revealed variation in circadian period length that correlated with temperature and aspects at the sites of plant origin, while the amplitudes of the rhythms correlated with soil composition. These data suggest that variation in environmental parameters exerts selection pressure on circadian rhythms and affects species adaptation (Dakhiya et al. 2017). A comparison of circadian clock rhythmicity and crop performance involving diverse barley lines (wild ancestors, landraces and breeding lines) under optimal and heat-stressed conditions revealed significant loss of thermal plasticity in circadian rhythms during domestication for output genes, while temperature compensation mechanisms was maintained in the core clock (Prusty et al. 2021). Domestication and artificial selection brought substantial changes in the developmental and circadian clock of plants possibly due to restructuring of plant metabolic timekeeping (Siqueira et al. 2023). Wild relatives of domesticated crops are an important genetic resource for identifying and exploiting such variations in crop breeding. Rapeseed and wheat cultivars, widely grown, also show variations in circadian periods (Rees et al. 2019).

Daily rhythms of gene expression are critical to ensure that biological processes occur at the optimal time of the day (TOD) for traits of agricultural significance. A gene expression study in rice grown in the field environment and temporally monitored for an entire growing season revealed that most genes have a TOD-specific expression over the developmental time course. The thermocycle genes have stronger cue for TOD expression than the photocyclic genes. The core circadian clock genes, except for two grass paralogs of *ELF3*, display consistent TOD expression over the season, while *ELF3* paralogs display distinct phases based on the interaction between thermo- and photo-cycles (Michael 2022).

The *PRRs* gene family in soybean exhibits rhythmic expression under LD and SD. The expression of most of the *PRR* genes is higher under LD in leaves. An investigating of the effects of natural variation in *GmPRR* alleles on soybean adaptation involving 207 germplasm accessions from China and USA. The majority of non-synonymous mutations in the coding region are associated with flowering time, while nonsense mutations—because of deletion of CCT domain—relate to early flowering (Wang et al. 2022b). Most of the Northeast China germplasm have haplotypes associated with early flowering, whereas those associated with late flowering are from lower latitudes. Thus, the *PRR* gene family in soybean provides a circadian clock-controlled flowering pathway and means to breed new germplasm adapted to specific daylength (Wang et al. 2022b).

Variation in circadian rhythm plasticity, denoting changes in period and amplitude under distinct temperatures, has been linked to both the maternal organellar genomes (plasmotype) and several nuclear loci in wild barley (Bdolach et al. 2019). Despite these findings, the molecular and biochemical basis of such cytonuclear interactions remain to be elucidated, as well as explored in other crops and models.

In short, assessing and exploiting functional allelic diversity in circadian rhythms and clock gene expression on a range of diversity panels of food crop germplasm may provide opportunity to tailor climate-resilient crops.

### Photoperiod (or temperature) × circadian clock × early flowering gene interaction to enhance crops range adaptation

Climate change and variability can affect plant productivity. The increase in temperature can accelerate the reproductive phase in plant life cycle, thus reducing harvests (Parthasarathi et al. 2022). It has been noted that plant development affected by photoperiod uses gene networks that respond to changes in the light/dark cycle length (Osnato et al. 2022). Such circadian clocks, which are important for photoperiod-dependent flowering in high latitudes, are also influenced by temperature changes, thus triggering seasonal responses (Oravec and Greenham 2022). Adapting crops to changing climate in high latitudes, as evidenced in rice and wheat, must consider the interactions between photoperiod, temperature, and water availability.

Transcriptome to research has provided insights on increased expression due to high temperature of core clock genes such as CCA1, *Gigantea-like* protein (*GI*), *PRR59*, *PRR73*, *PRR95*, and *HvLUX* (an ortholog of the *Arabidopsis* circadian gene *Lux Arrhythmo*, also known as *Phytoclock1*) in barley (Ford et al. 2016). *GI* and *PRR59* act rapidly when temperature increases, while both *CCA1* and *PRR73* respond when temperature decreases. The *elf3* variant of barley lacks the *GI* and *PRR* responses due to temperature changes, suggesting that *GI* and *PRR* depend on a functional *ELF3* (Ford et al. 2016). Thus, upregulation of core clock genes is pivotal in plants’ adaptation to temperature variations and the maintenance of circadian rhythms.

*HvLUX1* (under purifying selection) seems to underly the *early maturity 10* (*eam10*) mutant in barley (Campoli et al. 2013). The gene *eam10* leads to circadian defects and interacts with the pseudo-response regulator *Photoperiod-H1* (*Ppd-H1*) gene—a homolog of *Arabidopsis AtPRR7*—to speed flowering under both long and short photoperiods.

The emerging knowledge provides new insights for further genetic modification either through crossbreeding or genetic engineering. Modifying photoperiod through sexual hybridization, transgenics or gene editing will enlarge the range of adaptation in some daylength sensitive crops.

### Severely compromised clock gene expression and clock outputs can favor crop adaptation

For breeders, circadian genes and networks have not been a major focus of artificial selection efforts. Despite this, many agronomic traits such as sensitivity to light/dark cycles (Creux and Harmer 2019; Okada et al. 2017), abiotic stress tolerance (Bonnot et al. 2021; Sharma et al. 2022), flowering and maturation time (Creux and Harmer 2019), plant architecture (Rubin et al. 2018), and yield (Steed et al. 2021) are controlled or affected by components of the circadian clock. Thanks to advances in comparative omics, it has been shown that multiple components of the circadian clock are altered in cultivated crops, when compared to their wild relatives (McClung 2021; Steed et al. 2021). This has occurred even though the domestication and breeding processes were performed without having any explicit focus or knowledge about circadian rhythms.

**Fig. 2:**
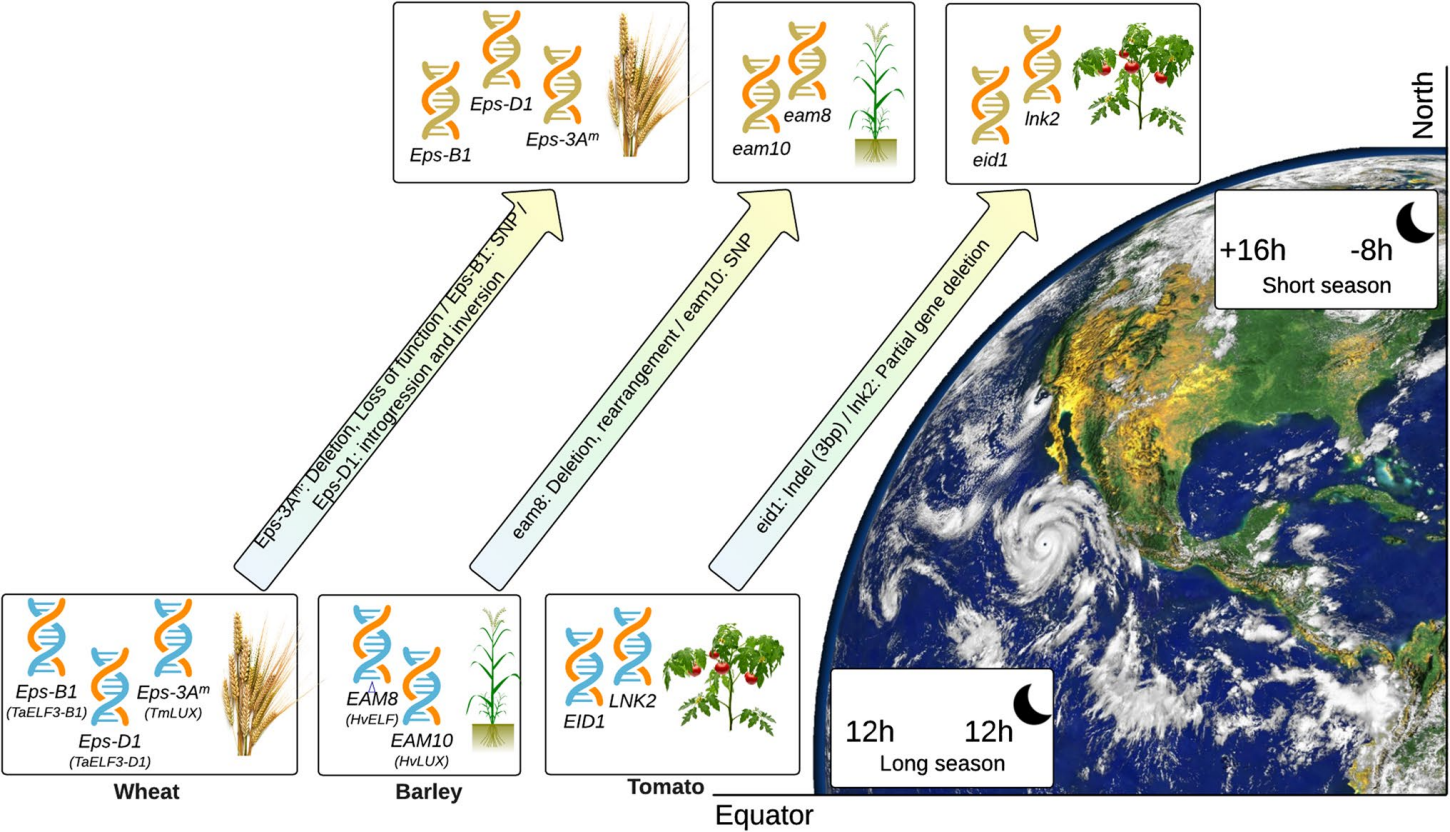
Effect of circadian allelic variations in the adaptation to different latitudes. Allelic variations linked to the adaptation for production in high-latitude (short-season and long-photoperiod) environments in *Solanum lycopersicum* (tomato); *Hordeum vulgare* (barley) and *Triticum sp.* (wheat)

Recent research has been focused on understanding and manipulation of circadian clock components as tools for plant breeding and adaptation. For example, in tomato, a 3 bp deletion in the gene *Empfindlicher imdunkelroten Licht protein 1* (*EID1*), which codes for a Phytochrome A-associated F-box protein, in addition to a near-complete deletion of *Night Light-Inducible and Clock-Regulated 2* (*LNK2;* orthologue to *AtLNK2*) produces a lengthening of the clock period (Müller et al. 2016, 2018). These modifications enabled tomatoes from the equatorial region in South America to adapt from day-neutral circadian rhythms to the longer photoperiod of Europe and Mesoamerica (Müller et al. 2016, 2018). Another example of modifications in the circadian components, that allowed adaptation to high-latitude short-season regions, are mutations in the *early maturity* (*EAM8*) gene from barley (Faure et al. 2012) and in the *earliness *per se (*Eps3*) from wheat (Gawroński et al. 2014), which are homologs of the *Arabidopsis thaliana* EC genes, *ELF3* and *LUX,* respectively (Fig. 2). These mutations in barley and wheat not only affect flowering time but also alter the circadian amplitude, period and phase (Faure et al. 2012; Gawroński et al. 2014). In a similar manner, allelic variations in *O. sativa* circadian heading-date genes (*Hd* genes not found in *Arabidopsis*), *Grain Number, Plant and Heading Date 7* (*Ghd7*), *Heading Date 1* (*Hd1*), *Early Heading Date 1 (Ehd1)* and *Early Heading Date 4* (*Ehd4*) have contributed to adaptation of rice to higher latitudes (Cui et al. 2020; Gao et al. 2013, 2014; Zhao et al. 2015; Zheng et al. 2016).

In *Arabidopsis*, multiple circadian genes are involved in tolerance to abiotic stress. *CCA1* regulates reactive oxygen species (ROS) homeostasis, where its overexpression produces an increased tolerance to ROS-generating agents; whereas loss-of-function mutations in *CCA1* and *LHY* lead to hypersensitivity (Lai et al. 2012). *TOC1*-silenced plants display improved tolerance and survival under drought, while its overexpression leads to an increase in water loss and a decrease in survival rate under drought (Legnaioli et al. 2009). Triple mutants for *PRR5, PRR7* and *PRR9* possess higher tolerance to drought and saline stress (Nakamichi et al. 2009). Mutant plants in the *GI* gene are more tolerant to salt and have a higher survival rate under drought and oxidative stress (Fornara et al. 2015; Kim et al. 2013). In rice, overexpression of the clock component *OsPRR73* confers increased salt tolerance and reduced cellular Na^+^ accumulation through the transcriptional repression of *HKT2;1,* a plasma membrane Na^+^ transporter, a gene negatively related to stress tolerance (Wei et al. 2021). It has been proposed that increased tolerance to abiotic stress, due to the mutation or overexpression of circadian components, is not because of the changes in the clock oscillation, but instead due to direct effects on genes involved in the response to abiotic stress (Grundy et al. 2015). Thus, circadian molecular components are emerging as potential agronomic and biotechnological targets for breeding crops better adapted to changing environments.

### High-amplitude rhythms for better adaptation under harsh conditions

Plants, as sessile organisms, are continuously exposed to multiple and variable changes in their environment. The circadian clock is a key component in the response to these changes. While the circadian phase and period are likely important for adaptation to environments with different light and temperature cycles, the robustness in the circadian amplitude seems to be related to the resilience to environmental changes. For example, the quintuple *Arabidopsis* mutant *cca1/lhy/rve468* (*quint*)—which possesses normal phase and period but reduced amplitude—has a robust circadian rhythm under optimal temperature conditions. In contrast, when *quint* mutant plants are grown in physiological non-optimal temperatures they display a pronounced arrhythmic behaviour (Shalit-Kaneh et al. 2018). Similarly, *quint* plants also have a fast reduction in the amplitude and arrhythmic behaviour in constant light and a reduced tolerance to genetic perturbation compared to wild-type plants (Shalit-Kaneh et al. 2018). It has also been discovered that narrowing diurnal temperature amplitude can negatively influence crop growth, illustrating that the amplitude of the environmental input affects the molecular circadian amplitude, ultimately affecting crop performance and yield (Shalit-Kaneh et al. 2018; Sunoj et al. 2020, 2016). Thus, a high-amplitude circadian rhythm could maintain a more robust rhythm in adverse and changing conditions, helping to cope the effect of harsh environmental conditions.

However, it is not fully elucidated how the amplitude of circadian rhythm affects adaptation to new environments or the tolerance/resilience to harsh environmental conditions. For example, it has been suggested that the disruption of the circadian rhythm could be beneficial to cope with cold stress, in which some circadian components increase the amplitude while others reduce it (Gil and Park 2019). This could reveal that not only amplitude is an important factor in the resilience to changes in the environment, but also the plasticity of the rhythm itself.

### Altered circadian rhythms and clock genes contribute to heterosis

#### Biomass and defense heterosis in *Arabidopsis*

Heterosis or hybrid vigor refers to the superior performance an F_1_ hybrid over its parents. Polyploidy (whole genome duplication) provides evolutionary advantages for plant growth, development, and adaptation. Some *Arabidopsis* allotetraploids and hybrids relative to their diploid parents (*Arabidopsis thaliana* and *A. arenosa*), can display vigour for morphological traits. Epigenetic modification of the circadian clock genes *CCA1*, *LHY* and their reciprocal regulators *TOC1* and *GI* leads to changes in the expression of downstream genes and pathways. Epigenetic repression of *CCA1* and *LHY* during the day induces *TOC1*, *GI,* and downstream genes harboring *CCA1* binding site (CBS) 13. Allotetraploids and F_1_ hybrids produce more chlorophyll and starch than parents in the same environment. Single *cca1* mutants, double *cca1 lhy*, and daily repression of *cca1* in *TOC1:cca1*-RNAi transgenic plants, led to increased expression of downstream genes, chlorophyll, and starch content. Notably, constitutive expression of *CCA1* or ectopically expressed *TOC1:CCA1* had opposite effects. These studies suggest that hybrids and allopolyploids gain advantages via circadian-mediated physiological and metabolic pathways that lead to growth vigor and increased biomass (Ni et al. 2009). Parent-of-origin effects on biomass heterosis are associated with altered *CCA1* expression amplitudes, which are associated with methylation levels of CHH (where H = A, T, or C) sites in the promoter region. The direction of rhythmic expression and hybrid vigor is reversed in reciprocal F_1_ hybrids involving mutants defective in the RNA-directed DNA methylation but not in maintenance methylation pathways. Such parent-of-origin effects on circadian regulation and heterosis is established during early embryogenesis and maintained throughout growth and development (Ng et al. 2014).

Plant immunity often causes growth-defense trade-offs. *CCA1* confers heterosis for bacterial defense in hybrids without yield penalty, and even enhances the growth heterosis of hybrids under pathogen infection in *Arabidopsis*. Its genetic perturbation abrogated heterosis for both defense and growth in hybrids. The improved heterosis for defense and growth of hybrids at different time points (dawn, dusk) of the day is achieved when both resistance and growth are the most effective and have the least fitness cost. Plants promote defense heterosis prior to salicylic acid burst by rhythmically enhancing growth vigor during infections in a diurnal manner (Yang et al. 2021).

#### Yield heterosis in grain crops

A comparative assessment of transcript profiles of super-hybrid rice ‘LY2186’ and its parents at multiple time points for 2 day/night cycles identified 1552 rhythmic differentially expressed genes, RDGs (Li et al. 2021). The day-phased RDGs were associated with photosynthesis and the night-phased RDGs were involved in stress response, the major contributor to heterosis in ‘LY2186’. The circadian-related RDGs were core components in both phases (day and night) and primarily regulate downstream genes involved in photosynthesis, starch synthesis, plant hormone signal transduction, and other pathways. RDGs (72% of 282 RDGs) mapped onto yield-related QTL could be considered as candidate genes, which after functional validation, may be deployed in hybrid rice breeding (Li et al. 2021). An integrative profiling of metabolome and proteome in maize hybrids and their inbred parents revealed diurnal regulation of several metabolites and proteins. Also, key enzymes in photosynthetic and photorespiratory pathways and metabolites in C assimilation had nonadditive abundance and mild metabolic heterosis in hybrids. Amino acids display negative mid-parent heterosis (MPH), while sugars, alcohols, and nucleotide metabolites had positive MPH. The daily changes in metabolites and proteins, though small, may accelerate hybrid growth. Photosynthetic pathway metabolites have positive MPH, whereas photorespiratory pathway metabolites have negative MPH, which correspond to nonadditive protein abundance and key enzyme activities in the respective pathways (Li et al. 2020).

Greater levels of C fixation and starch accumulation are associated with altered temporal gene expression of diurnally upregulated *ZmCCA1a* in maize. *CCA1a* homologs, *ZmCCA1a* and *ZmCCA1b*, are diurnally upregulated in hybrids. *ZmCCA1* expression complements the *cca1* mutant phenotype in *Arabidopsis*. *ZmCCA1b* overexpression disrupts circadian rhythms and biomass heterosis due to reduced chlorophyll content and plant height. Reduced stem height results from reduced node elongation but not the total number of nodes. The height phenotype is less severe in the field than in greenhouse. Enhanced light and/or metabolic activities in the field compensates for altered circadian regulation, and activation of morning-phased genes in the hybrids promotes photosynthesis and growth vigor (Ko et al. 2016).

### Circadian rhythms and clock genes mediating agronomic traits and abiotic stress adaptation

Transition from vegetative to reproductive phase, photoperiod-induced flowering, plant height, biomass and pod/seed traits, and storage roots in vegetatively propagated crops or tuberization are major agronomic traits influencing productivity of food crops. In addition to trait-responsive genes (i.e., core genes having identifiable direct impact on trait phenotype), several of these traits are also influenced by variation in circadian rhythms and clock genes.

#### Agronomic traits

Transition from vegetative to reproductive stage is critical to seed yield. Photoperiod pathways regulate flowering in rice, by synchronizing the exogenous environmental and the endogenous signaling cascades of photoreceptors, the circadian clock, and floral integrator genes. A diurnal rhythmic expression of *OsLUX* with the peak at dusk promoted flowering via the expression of genes associated with the circadian clock and the output integrators of photoperiodic flowering. *OsLUX* together with *OsELF4a* and *OsELF3a* or *OsELF3b* form two evening complexes (ECs), of which *OsLUX-OsELF3a-OsELF4a* predominantly promote photoperiodic flowering. *OsELF4a* alone also promotes flowering. Loss of *OsELF4a*, unlike *OsLUX*, marginally influences flowering under short day (SD), but markedly delays flowering under long day (LD) conditions (Cai et al. 2022).

Rice yields are predominantly determined by tillering. A regulatory loop involving the circadian clock, sugar, and strigolactone (SL) pathway regulates tiller bud and panicle development. *OsCCA1*, the circadian clock gene, positively regulates expression of SL pathway genes, *OsTB1*, *D14* and *IPA1* to repress tiller-bud outgrowth. *OsCCA1* overexpression or downregulation, respectively, reduces or increases tiller numbers, while manipulating *OsPRR1* expression causes opposite effects. *OsCCA1* also regulates *IPA1* expression to mediate panicle and grain development. *OsTB1*, *D14,* and *IPA1* act downstream of *OsCCA1*. Sugars affect *OsCCA1* negatively in roots and tiller buds but positively in the shoots to promote tiller-bud outgrowth. The circadian clock integrates sugar responses and the SL pathway to regulate tiller and panicle development (Wang et al. 2020a).

Optimizing crop duration to the available growing season is a major breeding target to match crop adaptation with the target environment. Variation in circadian-clock associated genes affects flowering in winter wheat cultivars in United Kingdom and European (Wittern et al. 2023). Early flowering* 3* (*ELF3*) is a candidate gene for earliness per se (Eps) at D1 and B1 loci in wheat genome. A SNP within the coding region of *TaELF3-B1* is the causal polymorphism at the *Eps-B1* locus. Deletion of the *Eps-D1* locus encompassing *TaELF3-D1* is the basis of an allele that lies within an introgression region containing an inversion relative to the Chinese Spring D genome. Circadian rhythm is severely disrupted in emmer wheat *(T. turgidum*) cv. Kronos that carries loss-of-function alleles at *TtELF3* locus, while loss of *LUX* in bread wheat, an *Arabidopsis* orthologue, also severely disrupts circadian rhythms. Thus, both *ELF3* and *LUX* are part of the wheat circadian oscillator, where they integrate the wheat EC (Rees et al. 2022; Steed et al. 2021). Indeed, there exists sufficient allelic diversity within the three wheat *ELF3* homoeologous to apply selection; i.e., delay or advance flowering, without adverse pleiotropic alterations to circadian rhythms in wheat (Wittern et al. 2023).

The ‘*j*’ allele in soybean delays flowering and enhances yield of long-juvenile soybean cultivars under SD conditions. ‘Huaxia-3’ (HX3) is a long-juvenile soybean cultivar with a loss-of-function allele *j* for the *J* gene. Transcriptome analysis of HX3 relative to the transgenic line overexpressing *J* gene unfolded 31 and 2311 differentially expressed genes, respectively, in HX3 under SD and LD conditions, with significantly enriched circadian rhythm pathway in HX3 under SD. *GmELF3a* and *FT* genes, *GmFT2a* and *GmFT5a,* were downregulated, while *GmFT4* was upregulated under SD in HX3. *FT* homolog *GmFT4*, in contrast, was downregulated while *GmFT1a* was upregulated under LD in HX3, thereby suggesting that *FT* homologs may cause delay in flowering of long-juvenile soybean under SD (Sapey et al. 2022). A previous report using a soybean hairy root expression system to monitor endogenous circadian rhythms and the sensitivity of circadian clock to environmental stimuli revealed that a quadruple mutant deficient in *GmLCLs* orthologs resulted in extreme short-period circadian rhythm and late flowering in soybean. Thus, morning phased *GmLCLs* act constitutively to maintain rhythmicity, while their absence delays vegetative to reproductive phase transition in soybean (Wang et al. 2020b).

Plants with indeterminate inflorescences produce more flower structures than those with determinate type inflorescences. Decoupling floral primordia initiations from their maturation into grains would mean more resources available for the plants to invest in other important physiological functions. Flowering time genes in barley regulate initiation of floral primordia, while floral growth is specified by light signaling, chloroplast, and vascular development orchestrated by *CCT MOTIF FAMILY 4* (*HvCMF4*), which is expressed in the inflorescence vasculature. Mutations in *HvCMF4* increase primordia death and pollen failure by reducing rachis greening and limit energy supply to floral tissues. *HvCMF4* senses light and connects with vascular-localized circadian clock to coordinate floral initiation and survival. Pyramiding beneficial alleles for both primordia number and survival may increase grain production in barley, caused by erratic stresses due to climate change and variability (Huang et al. 2023).

#### Abiotic stress adaptation

**Fig. 3:**
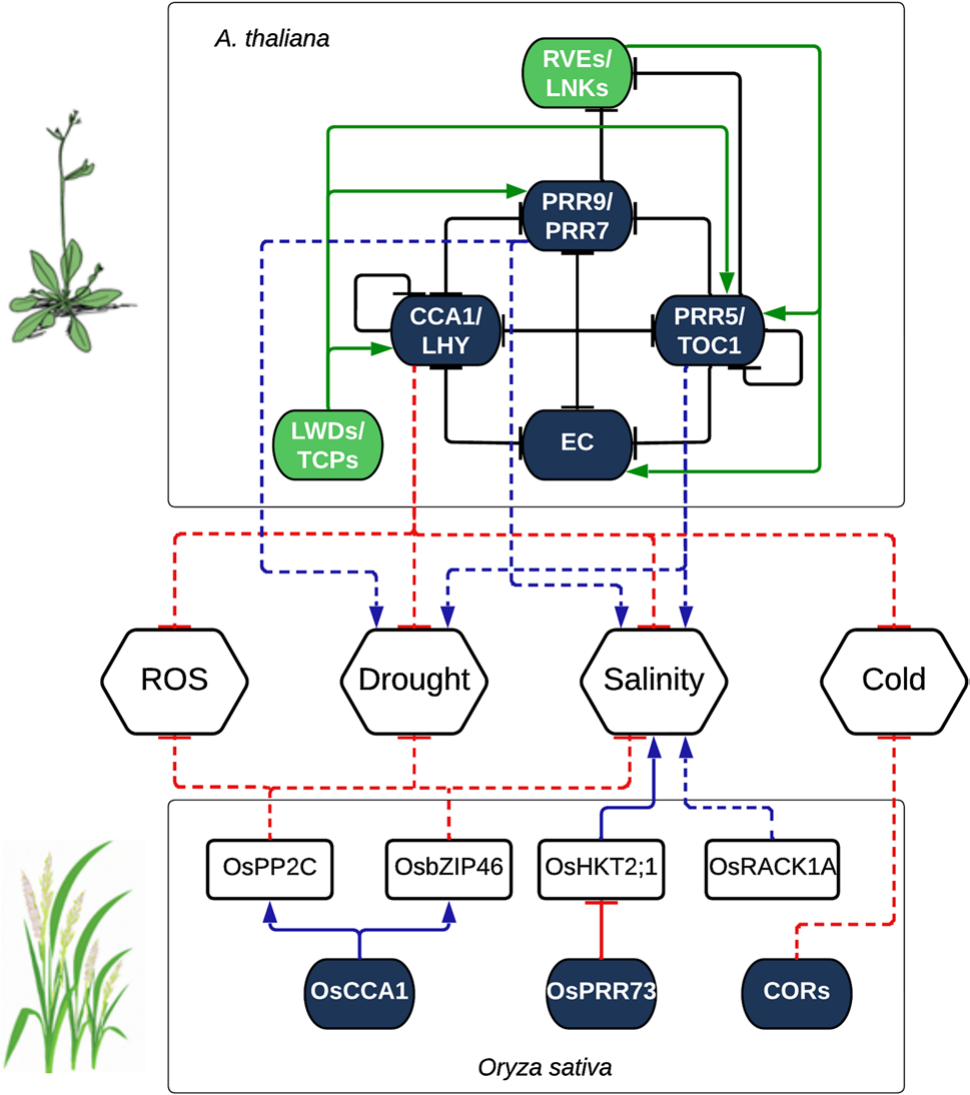
Circadian component effects under abiotic stress condition. This figure represents the effect of the mutation/silencing or overexpression of the circadian genes in the tolerance to different abiotic stresses. Solid lines represent direct regulatory effects while dashed lines represent indirect (or possibly direct but not experimentally determined) effects. Black lines ending with dashes represent the transcriptional repression of the circadian oscillator components. Green lines ending with arrows represent the transcriptional activation of the circadian oscillator components. Red lines ending with dashes represent transcriptional repression from or to the inputs and outputs in regard to the circadian oscillator. Blue lines ending with arrows represent transcriptional activation from or to the inputs and outputs regarding the circadian oscillator. The circadian repressor components in Arabidopsis are Circadian Clock Associated 1 (CCA1); Late Elongated Hypocotyl (LHY); Timing of CAB2 Expression 1 (TOC1); Pseudo-Response Regulator 9 (PRR9), PRR7 and PRR5; evening complex (EC). The circadian activation components in *Arabidopsis* are Reveille (RVEs); Night light-iducible and clock-regulated (LNKs); Light-regulated WD (LWDs); Teosinte Branched1-Cycloidea-PCE (TCPs). In Rice, OsCCA1 seems to transcriptionally activate protein phosphatase 2C (OsPP2C) and leucine zipper 46 (OsbZIP46), which have been shown to cope with the negative effects of ROS, drought, and salinity. OsPRR73 inhibit the High-affinity K + transporter 2;1 (OsHKT2;1) which is a negative factor in conferring tolerance to saline conditions. Similarly, the receptor for activated C kinase 1A (OsRACK1A) is circadian regulated and has been shown to have a negative effect increasing the sensitivity to salinity

Several clock genes that impact circadian rhythms and abiotic stress adaptation are summarized herewith in diverse crops (Fig. 3). Melatonin (*N*-acetyl-5-methoxy-tryptamine) is a key hormone that functions as indole-3-acetic acid like hormones in mammals and plants, thereby regulating gene expression affects circadian rhythms, plant growth and development, and stress tolerance (Fan et al. 2018). Abiotic stress impacts the expression pattern of key melatonin biosynthetic genes. In rice, drought stress under light and dark conditions damages circadian clock genes, *tryptophan decarboxylase* (*TDC*), *tryptamine 5-hydroxylase* (*T5H*), and *serotonin N-acetyltransferase* (*SNAT*), but retains the functionality (circadian rhythm) of *N-acetylserotonin O-methyltransferase* (*ASMT*) gene in wild-type (WT) plants. Downregulation of *ASMT* expression in *OsGI* mutation suggests the involvement of the melatonin biosynthetic pathway in the *OsGI*-mediated circadian regulation. Thus, there exists a coordination between circadian rhythms and abiotic stress with respect to melatonin biosynthesis in rice (Ahn et al. 2021). However, more focused research on the role of melatonin on plant circadian clock may provide evidence-based mechanistic connection between melatonin and the circadian clock.

Drought accelerates leaf senescence which reduces canopy size, loss in photosynthesis, and reduced yields, while drought-induced delayed leaf senescence (i.e., stay green) contributes to crops fitness and productivity (Rivero et al. 2007). Variation in internal signals and environmental factors contribute to initiation and progression of leaf senescence (Guo et al. 2021; Zhang et al. 2021). *ZmVQ52* is mainly expressed in maize leaves and is involved in the circadian pathway genes and photosynthetic pathways. WRKY family proteins (ZmWRKY20, ZmWRKY36, ZmWRKY50, ZmWRKY71) interact with *ZmVQ52*. *ZmVQ52* overexpression accelerated pre-mature leaf senescence in *Arabidopsis*. Several leaf senescence genes in *ZmVQ52* overexpression lines were upregulated by premature leaf senescence in *Arabidopsis* (Yu et al. 2019).

A large number of heat stress-responsive genes exhibit diurnal oscillation in *Arabidopsis*. Wild-type and mutants of the circadian clock genes (*CCA1*, *LHY*, *PRR7*, *PRR9*) exposed to heat (37 °C) and moderate cold (10 °C) during Zeitgeber (a rhythmically occurring natural phenomenon which acts as a cue in the regulation of the plant circadian rhythms) time ZT1 (early morning) and ZT6 (afternoon) revealed thousands of genes that were differentially expressed in response to temperature, the time of the day, or the clock mutation. In the morning, ~ 30% more genes were differentially expressed than in the afternoon. Heat stress significantly perturbed the transcriptome. Seventy percent of ~ 3000 DEGs showed time of the day (ZT1 or ZT6) occurrence of the transcriptional response. Changes in the magnitude of transcriptional response were detected in ~ 1400 DEGs shared between ZT1 and ZT6. About 2% of all DEGs had differential responses to temperature stress in the clock mutants. The clock genes *CCA1* and *LHY* seem to have a more profound role than PRR7 and *PRR9* in modulating heat stress responses during the day. Thus, time of the day plays a significant role in quantifying heat stress-responsive transcriptomes (Blair et al. 2019).

RACK1 is a WD40 type protein involved in multiple signaling pathways, conserved from prokaryotes to eukaryotes, and is involved in diverse biological functions and stress tolerance in plants (Adams et al. 2011; Zheng et al. 2017). *OsRACK1A* is a circadian rhythm gene involved in regulating salt tolerance in rice. *OsRACK1A*-suppressed transgenic rice compared to WT maintained low Na^+^ and high K^+^ concentrations, accumulate significantly more ABA and its transcripts and stress-inducible genes. Many stress-related genes, including AP2/ERF, were upregulated in *OsRACK1A*-suppressed transgenic rice (Zhang et al. 2018a). Another clock component, *OsPRR73,* positively regulates salt tolerance in rice by recruiting HDAC10 which transcriptionally repressed OsHKT2; 1, thus reducing cellular Na^+^ accumulation. *osprr73* null mutants accumulated significantly higher levels of reactive oxygen species and sodium ions, which resulted in reduction in grain size and yield under salt stress (Wei et al. 2021).

Overall, circadian rhythmicity and clock genes that respond to abiotic stress, in turn interact with stress response genes to confer tolerance, providing broadened opportunities to develop more sustainable and productive cereal crops. In addition, several candidate clock genes with differential expression levels were identified as potential target for functional validation, for example, *StGI*, *StPRR*, and *StEFM* affecting tuberization in potato (Niu et al. 2022); *COR* expression in day and night impacting cold tolerance (Lu et al. 2021) and variable expression levels of morning (*OsPRRs*, *OsLHY*, *OsZTL1*) and evening (*OsTOC1*, *OsGI*, *OsELF3*) circadian clock genes in response to drought stress in rice (Li et al. 2017); *HvELF3* expression impacting phase and shape of the clock and stress response (Habte et al. 2014) and variable expression of core clock genes (*CCA1*, *GI*, *PRR59*, *PRR73*, *PRR95*, and *LUX*) at higher temperatures (Ford et al. 2016) in barley; or circadian clock genes impacting significantly phase and amplitude in root and shoot under varying water regimes in the monocot model plant *Brachypodium distachyon* (Gombos et al. 2023).

### Transgenic modification or genome editing to alter circadian rhythms to enhance abiotic stress adaptation and crops productivity

Modifying the circadian clock should be a breeding target to develop cultivars with higher buffering capacity to environmental stresses. Transgenic rice (japonica variety Taipei309) overexpressing *CCA1* under the control of TOC1 promoter and containing RNAi construct A that follows circadian rhythm in progeny (T_1_, T_2_) plants has a detrimental effect on phenology and seed yield related traits over WT. *CCA1* repression under the control of TOC1 promoter and containing RNAi constructs B and C that follows circadian rhythm in progeny plants significantly improve phenology and seed yield related traits over WT. Furthermore, RNAi construct C containing *CCA1* derived from the 3’ terminal region of *CCA1* generates plants with better phenology than construct B obtained from 5’ terminal region of *CCA1*. The progeny plants did not differ in chlorophyll content but had distinct rhythmicity, relative to *CCA1* expression. Thus, *CCA1* under the control of TOC1 promoter offers promise for development of crop cultivars better adapted to variable climates (Chaudhary et al. 2019).

CRISPR-Cas9 system has shown promise for plant breeding (Haque et al. 2018). Protein–protein interaction networks of differentially expressed proteins offer opportunities for better understanding of the organism response at the whole proteome level (Zou et al. 2018). A comparative proteomic analysis between CRISPR-Cas9 generated *OsPYL9* loss-of-function mutants and WT at the whole proteome level revealed that CRISPR mutants had enhanced drought tolerance and improved grain yield under optimal and water-deficit conditions. Multiple proteins were differentially expressed (184 upregulated and 140 downregulated), with most of the upregulated DEPs related to circadian clock rhythm, drought response, and antioxidant activities in the mutants. DEPs were involved in response to abiotic stimulus and abscisic acid-activated signaling pathways. GIGANTEA, Adagio-like, and pseudo-response regulator proteins revealed higher interaction in protein–protein interaction (PPI) network. Both DEPs and higher PPI network in *OsPYL9* mutant affect circadian rhythmicity, and a potential genetic resource and biomarker to breed for increased drought tolerance and improved yield in rice (Usman et al. 2020).

Overall, the results summarized in this section indicate the potential of genetic engineering for modifying circadian rhythmicity to enhance stress tolerance and productivity in food crops.

## Managing circadian clock for breeding all seasons’ crops

The circadian clock that is based on interlocked negative loops of transcriptional activators and repressors is crucial for adjusting crop growth. Genes that maintain the plant circadian clock “ticking” (e.g. morning genes: *CCA1*, *LHY*; mid-day genes: *PRR9*, *PRR7*, *PRR5*; evening genes: *TOC1*, *ELF3*, *ELF4*, *LUX*) are supported by evidence in model plant *Arabidopsis* and other crops. Clock genes impacting biological rhythms and associated with plant growth, development, reproduction, and stress tolerance, have been identified in staple crops. Likewise, the role of phytohormones in mediating clock and stress-responsive gene expression is known. Maintaining the intricate interactions between circadian rhythms and clock genes‒phytohormones‒trait/stress-responsive genes may open new paths to tailor crops adapted to external environmental cues. Crop adaptation by changing flowering time is a good example of the potential of clock genes, where loss of some segments of circadian clock genes facilitated adaptation of tomatoes to distant growing habitats far from its center of origin (Müller et al. 2016, 2018).

**Fig. 4:**
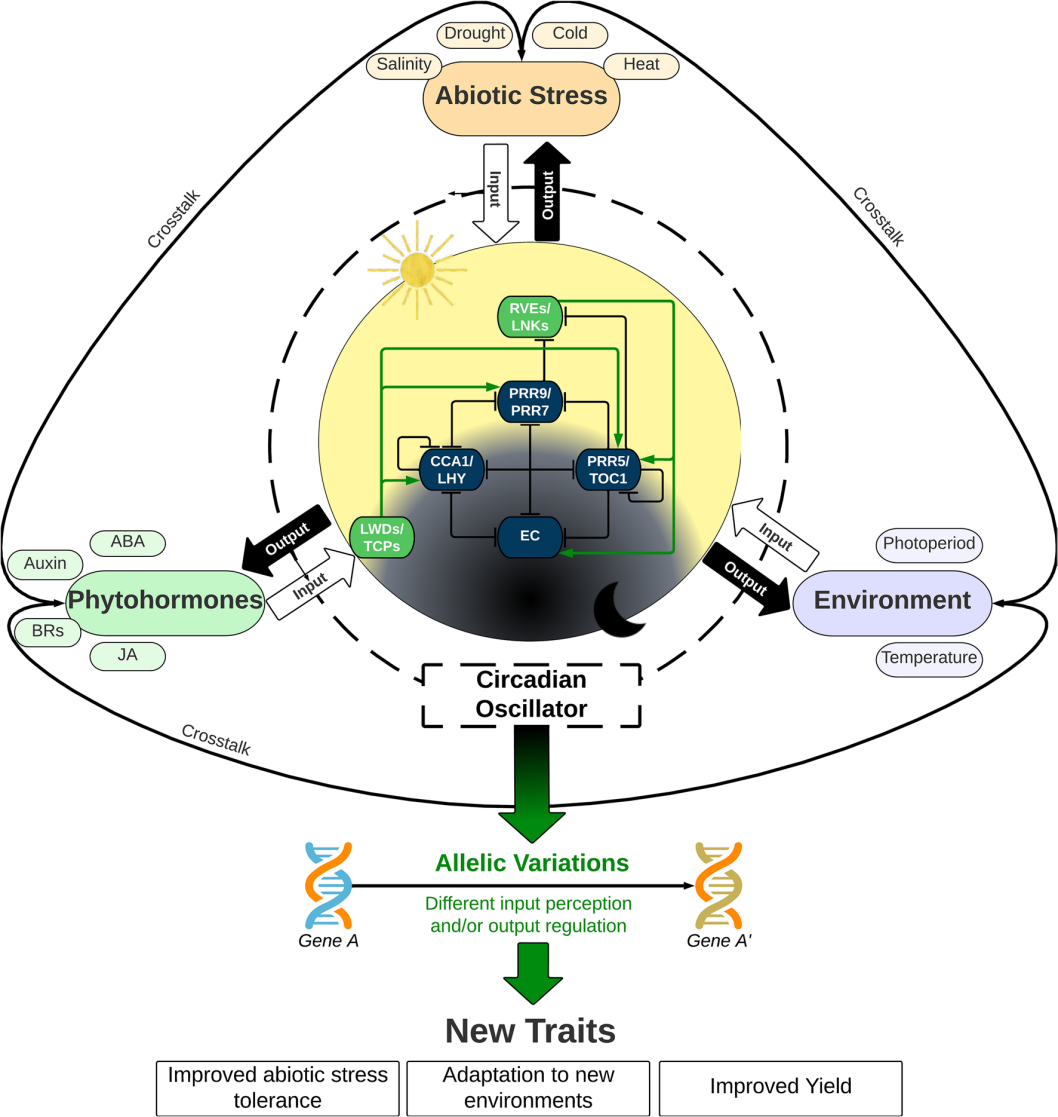
The circadian oscillator in plants: integrating environmental and physiological cues to improve adaptation and resilience. The intricate circadian oscillator in plants continually integrates environmental and physiological cues, including signals from phytohormones, natural environments, and abiotic stress conditions. The oscillator responds to these inputs with coordinated output signals, allowing the plant to adjust its physiology and behaviour accordingly. Recent research has revealed that natural allelic variations or induced mutations in genes involved in the regulation of the circadian oscillator can significantly impact how plants perceive inputs or regulate outputs, ultimately leading to improved adaptation to new environments, enhanced tolerance to abiotic stress, and increased crop yields. This figure highlights the importance of understanding the genetic and molecular mechanisms underlying the circadian oscillator’s regulation and how they can be manipulated to improve plant performance and resilience

The knowledge emerging on circadian clock molecular functioning can facilitate breeding of all seasons’ crops (Fig. 4). However, pursuit of such an approach poses a significant breeding challenge due to various factors; namely, (i) harnessing circadian oscillator genes that confer variation in biological rhythms in crop genepools, (ii) significant bottlenecks to establishing cost-effective high-throughput phenomics to assess variation in functioning of circadian oscillators in early breeding generations, (iii) possible negative trade-offs involving clock oscillator genes, phytohormones, abiotic stresses, and gene expression (i.e., trait- and stress-responsive genes), and (iv) identifying common genetic markers (e.g., SNPs, InDels and structural variation) linked with clock oscillators, phytohormones, and gene expression. Thus, assessing and exploiting functional allelic diversity in core oscillators as well as in trait-and stress-responsive genes can accelerate the development of climate-resilient productive and value-added germplasm for use in crop breeding.

## Data Availability

No new data were generated for preparation of this review paper. Data sharing not applicable to this article as no datasets were generated or analyzed during the current study.
